# The Melatonin Type 2 Receptor Agonist IIK7 Attenuates and Reverses Morphine Tolerance in Neuropathic Pain Rats Through the Suppression of Neuroinflammation in the Spinal Cord

**DOI:** 10.3390/ph17121638

**Published:** 2024-12-05

**Authors:** Yaswanth Kuthati, Chih-Shung Wong

**Affiliations:** 1 Department of Anesthesiology, Cathay General Hospital, Taipei 106, Taiwan; yaswanthk1987@gmail.com; 2 Graduate Institute of Medical Sciences, National Defense Medical Center, Taipei 280, Taiwan

**Keywords:** neuropathic pain, MOR tolerance, IIK7, neuroinflammation, MT2 receptor agonist

## Abstract

Background: Morphine analgesic tolerance (MAT) limits the clinical application of morphine in the management of chronic pain. IIK7 is a melatonin type 2 (MT2) receptor agonist known to have antioxidant properties. Oxidative stress is recognized as a critical factor in MAT. This study sought to assess the impact of IIK7 on the progression of MAT and its potential to reverse pre-existing MAT. Methods: Wistar rats underwent partial sciatic nerve transection (PSNT) surgery to induce neuropathic pain (NP). Seven days post nerve transection, we implanted an intrathecal (i.t.) catheter and linked it to an osmotic pump. Rats were randomly divided into the following groups: sham-operated/vehicle, PSNT/vehicle, PSNT/IIK7 50 ng/h, PSNT/MOR 15 g/h, and PSNT/MOR 15 g + IIK7 50 ng/h. We implanted two i.t. catheters for drug administration and the evaluation of the efficacy of IIK7 in reversing pre-established MAT. We linked one to an osmotic pump for MOR or saline continuous i.t. infusion. On the 7th day, the osmotic pump was disconnected, and 50 μg of IIK7 or the vehicle was administered through the second catheter. After 3 h, 15 μg of MOR or saline was administered, and the animal behavior tests were performed. We measured the levels of mRNA for Nrf2 and HO-1, pro-inflammatory cytokines (PICs), and the microglial and astrocyte activation in the spinal cord. Results: The co-administration of IIK7 with MOR delayed MAT development in PSNT rats by restoring Nrf2 and HO-1 while also inhibiting the microglial-cell and astrocyte activation, alongside the suppression of PICs. Additionally, a single injection of high-dose 50 μg IIK7 was efficient in restoring MOR’s antinociception in MOR-tolerant rats. Conclusions: Our results indicate that the co-infusion of ultra-low-dose IIK7 can delay MAT development and a high dose can reverse pre-existing MAT.

## 1. Introduction

Neuropathic pain, resulting from the injury or dysfunction of the peripheral or central nervous system (CNS), is a common symptom in numerous conditions such as human immunodeficiency virus (HIV) infection, stroke, chemotherapy, and surgical interventions [1]. Research indicates that 15–50% of individuals with neuropathic pain report experiencing allodynia, defined as pain caused by a non-painful stimulus, and hyperalgesia, characterized by an amplified pain response to a typically painful stimulus [2]. Glial–neuronal interactions link pain hypersensitivity and the persistence of chronic pain [3]. Glial cells initiate various signaling pathways that modulate pain processing at both spinal and supraspinal levels. The secretion of pro-inflammatory cytokines (PICs) and chemokines promotes the proliferation of pain [4]. Neuroinflammation is characterized by the activation of microglia and astrocytes, the generation of chemokines and cytokines, and the activation and infiltration of leukocytes [5]. Peripheral nerves, dorsal root ganglia, and the CNS often exhibit elevated levels of inflammatory mediators, including chemokines and cytokines, which are essential in the onset and persistence of chronic pain [6,7,8].

Current therapeutic strategies for chronic pain management, such as painkillers and physiotherapy, frequently prove to be insufficient and ineffective [9,10]. Neuropathic pain treatment often includes opioids such as morphine (MOR) [11]. However, prolonged exposure to MOR leads to MOR analgesic tolerance (MAT). The current literature indicates that MAT induces oxidative stress and diminishes endogenous antioxidant levels [12,13]. Researchers indicate oxidative stress as a critical contributor to MAT and dependency. [14]. Oxidative stress is known to activate microglial cells and astrocytes [14]. It is known that activated microglia and astrocytes play significant roles in MAT and suppressing glial activation attenuates MAT [15,16]. Researchers have postulated numerous methods to elucidate the direct and indirect activities of MOR that activate glial cells, including its binding to mu-opioid receptors, Toll-like receptor 4, ATP receptor P2X4, and other chemokine receptors, among others [17,18,19].

Melatonin (MLT) is crucial for the regulation of sleep and circadian rhythms. Neurological illnesses can benefit from the antioxidant and anti-inflammatory properties of MLT [20]. Researchers recognize MLT for its analgesic and neuroprotective properties [16,21,22]. Nonetheless, the therapeutic efficacy of MLT and its related processes remain under examination. Multiple MLT receptor agonists (e.g., Ramelteon and Tasimelteon) have received approval from the United States Food and Drug Administration (USFDA) for the management of sleep and depressive disorders. Certain drugs, including TIK-301 and piromelatine, have commenced clinical studies [23]. Nevertheless, MLT agonists have not yet received approval for the treatment of NP. Nonetheless, preclinical evidence indicates that MLT agonists provide significant potential in the treatment of NP [24,25,26,27,28]. Melatonin type 2 (MT2) receptors play a significant role in the modulation of pain pathways [29]. The activation of MT2 receptors can yield substantial analgesic effects. Although melatonin type 1 (MT1) receptors contribute to pain modulation, their effect is less significant than that of MT2 receptors. MT1 receptors are crucial in the regulation of circadian rhythms and several physiological processes [30]. IIK7 is a melatonin derivative that selectively interacts with the MT2 receptor [31]. IIK7 had demonstrated its sleep-enhancing qualities and ability to mitigate electromagnetic stress [32,33]. We previously demonstrated that IIK7 alleviates neuropathic pain by lowering the spinal ROS levels [34]. This preclinical study evaluated the impact of IIK7 on the following variables: (1) we evaluated the impact of IIK7 on the development of MAT through the co-infusion of 50 ng; (2) the capacity of IIK7 in restoring MOR antinociception was evaluated in MOR-tolerant rats with a high dose of IIK7, 50 μg; (3) the impact on the restoration of Nrf2 and HO-1 neuroprotective proteins, along with the effect on PIC levels, was evaluated; and (4) we examined the impact of IIK7 co-infusion with MOR on glial-cell and astrocyte activation.

## 2. Results

### 2.1. Co-Infusion of an Ultra-Low Dose of IIK7 Attenuates MOR’s Antinociceptive Tolerance in PSNT

**Figure 1:**
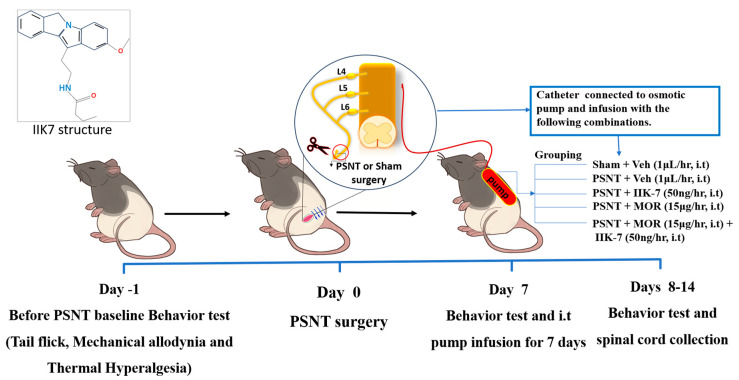
Impact of ultra-low dose IIK7 co-infusion on MOR tolerance development.

MOR tolerance was induced through i.t. MOR infusion at a rate of 15 μg/h over a duration of one week, as illustrated in Figure 1.

**Figure 2:**
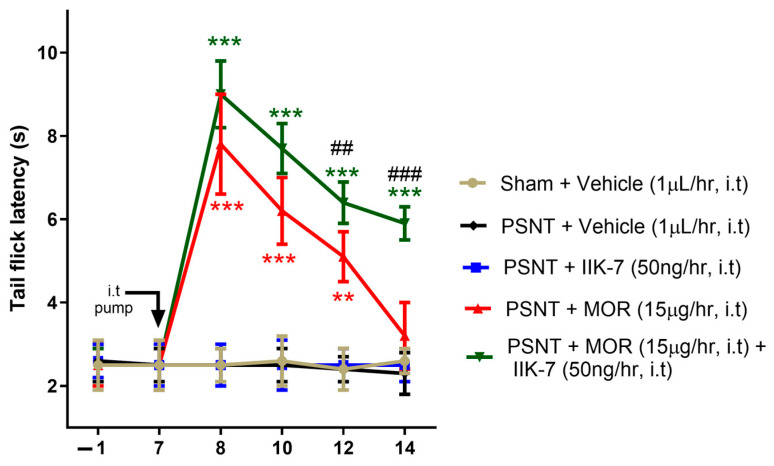
IIK7 co-infusion alleviates MOR-induced antinociceptive tolerance against acute pain. The baseline tail-flick response was assessed on Day −1 (one day prior to PSNT) and on Day 7 post PSNT. Following the baseline tail-flick measurements on Day 7, osmotic pumps were implanted, and the tail-flick response was assessed on Days 8, 10, 12, and 14 through constant i.t. delivery of the following: vehicle (1 µL/h), IIK7 (50 ng/h), MOR (15 μg/h), and a combination of MOR (15 μg/h) and IIK7 (50 ng/h) in both the sham and PSNT groups. Statistical significance levels are indicated as follows: ** *p* < 0.01, and *** *p* < 0.001, in comparison to the PSNT + Vehicle group. Statistical significance is indicated by ## *p* < 0.01, and ### *p* < 0.001 when comparing PSNT + MOR to PSNT + MOR + IIK7 (*n* = 6).

Figure 2 illustrates that the MOR infusion had its maximum antinociceptive effect on Day 8 post PSNT (i.e., Day 2 of the osmotic pump infusion). The development of MAT was evident by Day 14 (i.e., Day 5 of osmotic pump infusion), peaking between Days 12 and 14 (5–7 days of osmotic pump infusion). The tail-flick latency decreased from 7.9 to 1.5 s on Day 2 to 3.1 to 1.2 s on Day 7 of MOR infusion. The co-infusion of an ultra-low dose of IIK7 50 ng/h along with MOR significantly enhanced MOR’s analgesic effects, as evident from a tail-flick latency of 9.2 ± 1.2 s on Day 2 of infusion vs. 7.9 ± 1.5 s in MOR-infused rats. In addition, IIK7 attenuated the development of MAT, as evident from a tail-flick latency of 6.1 ± 1.1 s on the 7th day of infusion compared to the MOR-infused rats with a tail-flick latency of 3.1 ± 1.2 s.

### 2.2. The Effects of Continuous i.t. Administration of MOR, IIK7, and MOR + IIK7 on Mechanical Allodynia and Hyperalgesia

**Figure 3:**
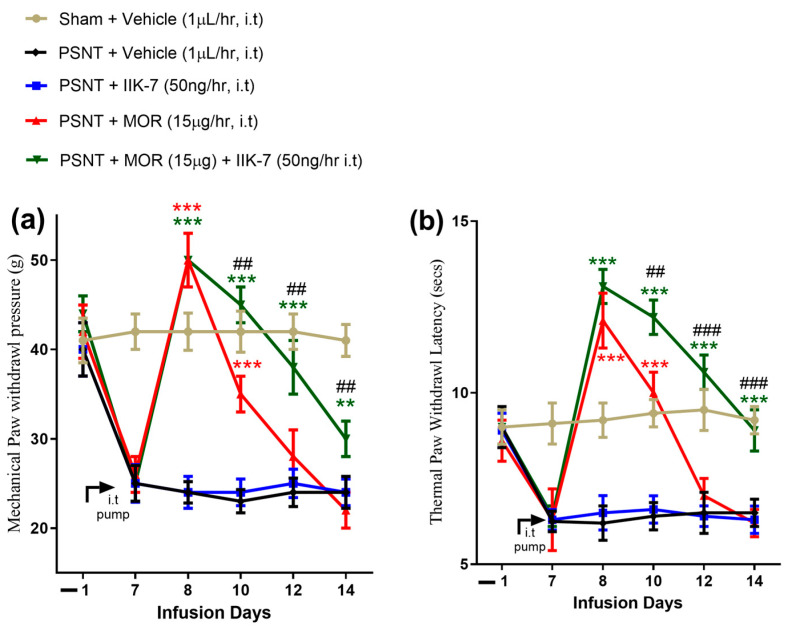
IIK7 co-infusion alleviates MOR-induced analgesic tolerance in neuropathic−pain rats. For the behavior tests, the baseline (**a**) mechanical paw withdrawal threshold and (**b**) thermal withdrawal latency were measured on Day −1 (1 day before performing PSNT) and Day 7 after PSNT. Following the baseline measurement on Day 7, osmotic pumps were installed, and mechanical paw withdrawal threshold and thermal paw withdrawal were evaluated on Days 8, 10, 12, and 14 through constant intrathecal infusion of the vehicle (1 µL/h), IIK7 (50 ng/h), MOR (15 μg/h), and MOR (15 μg/h) + IIK7 (50 ng/h) in the sham and PSNT groups. ** *p* < 0.01, and *** *p* < 0.001 compared to PSNT + Vehicle group. Statistical significance is indicated by ## *p* < 0.01, and ### *p* < 0.001 when comparing PSNT + MOR to PSNT + MOR + IIK7 (*n* = 6).

The sham rats exhibited a higher withdrawal threshold compared to the PSNT groups (41 ± 3 g vs. 25 ± 4 g; Figure 3b, yellow, red, green, blue, and black curvatures), indicating the successful induction of PSNT-related neuropathic pain in the nerve transected rats. Compared to the PSNT vehicle group, the infusion of IIK7 at a rate of 50 ng/h did not demonstrate significant antinociceptive effects (Figure 3a, blue-color curve). The i.t. administration of MOR at a dosage of 15 µg/h significantly decreased allodynia in the PSNT group in comparison to the vehicle-administered PSNT rats on the first day of i.t. infusion (50 ± 2.5 vs. 25 ± 3 g; Figure 3a, red-color curve and black curve). The paw withdrawal threshold (PWT) in the MOR group exhibited a significant decrease on Day 7 of infusion as a result of MAT, measuring 22 ± 2 g, which did not show a significant difference compared to the PSNT vehicle-infused group (Figure 3a, red-color and black-color curves). MOR co-infusion with IIK7 led to a notable increase in the PWT on Day 7, measuring 31 ± 5 g compared to 22 ± 2 g for MOR infusion alone, suggesting a decrease in MAT (Figure 3a, red-color and green-color curves).

The sham rats exhibited a higher withdrawal latency compared to the PSNT group (8.2 ± 0.5 s vs. 6.0 ± 0.8 s; Figure 3b, yellow, red, green, blue, and black curves), indicating the successful induction of PSNT-related neuropathic pain in the nerve-transected rats. Compared to the PSNT vehicle rats, the continuous i.t. infusion of IIK7 at a rate of 50 ng/h did not demonstrate significant antinociceptive effects (Figure 3b, blue curve). The i.t. administration of MOR at 15 µg/h significantly reduced hyperalgesia in the PSNT rats in comparison to the vehicle-administered PSNT rats on the first day of i.t. drug infusion (12 ± 1.5 vs. 6.0 ± 1.1 s; Figure 3a, red curve and black curve). The MOR-administered group exhibited a significant increase in thermal hyperalgesia on Day 7 of infusion as a result of MAT, with a paw withdrawal latency (PWL) of 6.0 ± 0.5 s, which did not show a significant difference compared to the PSNT vehicle-administered rats (Figure 3a, red-color and black-color curves). The co-infusion of MOR with IIK7 led to a notable increase in the PWL in comparison to the MOR administration alone on the first day (12.5 ± 0.5 s vs. 13.7 ± 0.8 s) and Day 7 (6.0 ± 0.5 s vs. 8.2 ± 0.3 s), suggesting a decrease in MAT (Figure 3a, red and green curves).

### 2.3. IIK7 Co-Infusion with MOR Reversed MOR-Induced Suppression of Nrf-2 and HO-1 Antioxidant Genes

**Figure 4:**
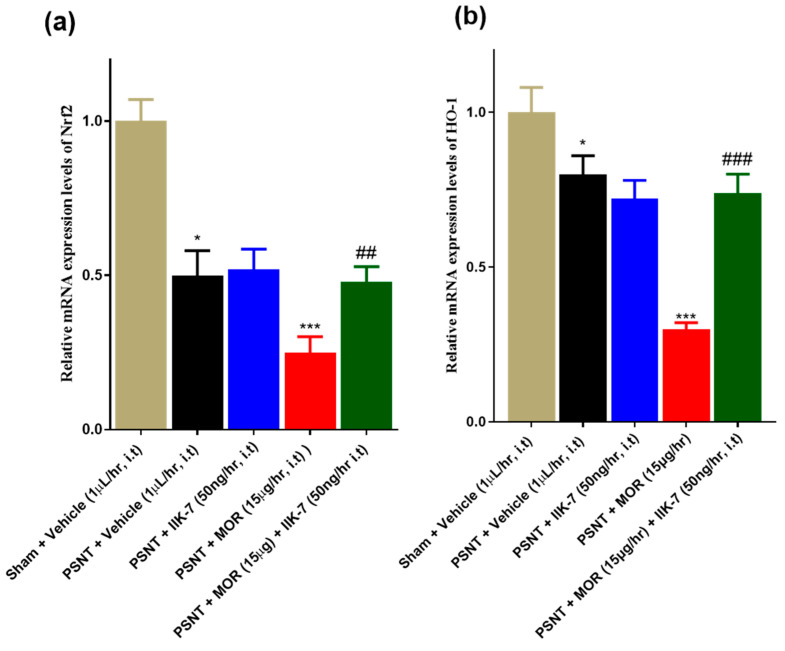
Relative mRNA expression levels of the neuroprotective proteins (**a**) Nrf2 and (**b**) HO-1 were assessed using RT-qPCR. The asterisk indicates a statistically significant difference among the following comparisons: sham + Vehicle versus PSNT + Vehicle, PSNT + Vehicle versus PSNT + IIK7, and PSNT + Vehicle versus PSNT + MOR. The symbol # denotes a statistically significant difference between PSNT + MOR and PSNT + MOR + IIK7. Significance levels are indicated as follows: * *p* < 0.05; ## *p* < 0.01; ###/*** *p* < 0.001. Six animals were included in each group (*n* = 6).

Figure 4a,b illustrate the mRNA levels of Nrf2 and HO-1 in the spinal cord of sham and PSNT groups infused intrathecally with the following combinations: vehicle alone, MOR, IIK7, and MOR + IIK7 over a duration of 7 days. The findings demonstrated a reduction in the levels of Nrf2 and HO-1 expression in the spinal cord of PSNT rats relative to the sham rats (Figure 4a,b, yellow and black bars). The IIK7 infusion alone did not affect the restoration of Nrf-2 and HO-1 expression in the PSNT group in comparison to the PSNT + Vehicle rats (Figure 4a,b, black-color and blue-color bars). MOR infusion markedly reduced Nrf2 and HO-1 expression in comparison to the PSNT + Vehicle infusion rats (Figure 4a,b, red bars). The combination of IIK7+MOR markedly enhanced Nrf-2 and HO expression in comparison to the MOR group (Figure 4a,b, green bars).

### 2.4. Co-Infusion of IIK7 with MOR Led to a Decrease in Pro-Inflammatory Cytokine Levels in PSNT Rats

**Figure 5:**
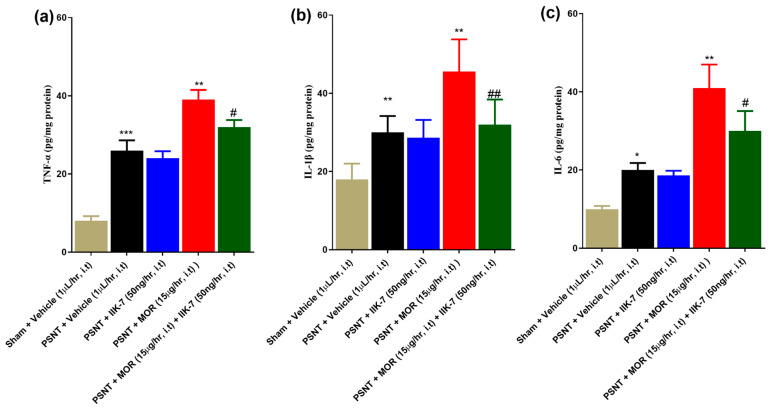
Impact of vehicle, PSNT, IIK7, or MOR or MOR + IIK7 infusion on pro-inflammatory cytokine concentrations in the spinal cord. PSNT surgery and MOR infusion significantly elevated all three PIC levels—TNF-α (**a**), IL-1β (**b**), and IL-6 (**c**)—in the spinal cord samples. The asterisks indicate statistically significant differences between the following comparisons: sham + Vehicle versus PSNT + Vehicle, PSNT + Vehicle versus PSNT + IIK7, and PSNT + Vehicle versus PSNT + MOR. # indicates a statistically significant difference between PSNT + MOR and PSNT + MOR + IIK7. Statistical significance levels are indicated as follows: */# *p* < 0.05; **/## *p* < 0.01; *** *p* < 0.001. Sample size consisted of 6 animals per group.

ELISA results showed that PSNT rats secreted higher levels of the PICs TNF-α, IL-1β, and IL-6 when compared to the sham group (Figure 5a–c, yellow and black bars). The administration of IIK7 alone did not significantly affect the levels of PICs (Figure 5a–c, red bars). However, the co-infusion of IIK7 significantly reduced the secretion of all three PICs (Figure 5a–c, green bars).

### 2.5. IIK7 Reverses MOR Antinociceptive Tolerance in PSNT Rats with Pre-Established MAT

**Figure 6:**
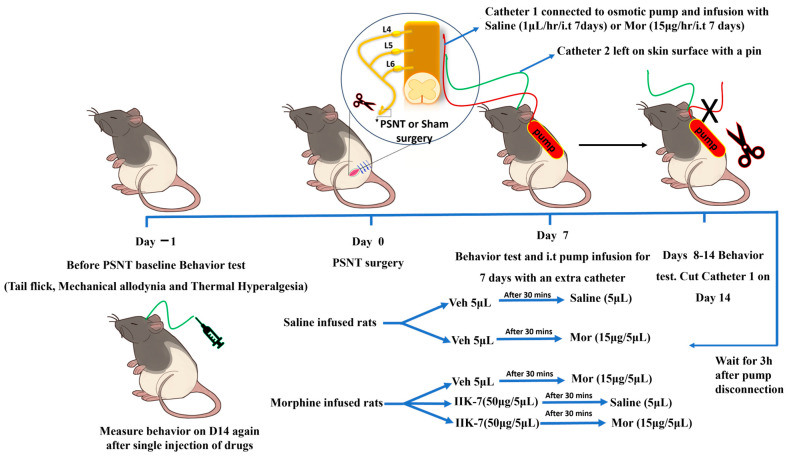
Impact of a high-dose IIK7 single injection on rats with pre-established MOR tolerance.

**Figure 7:**
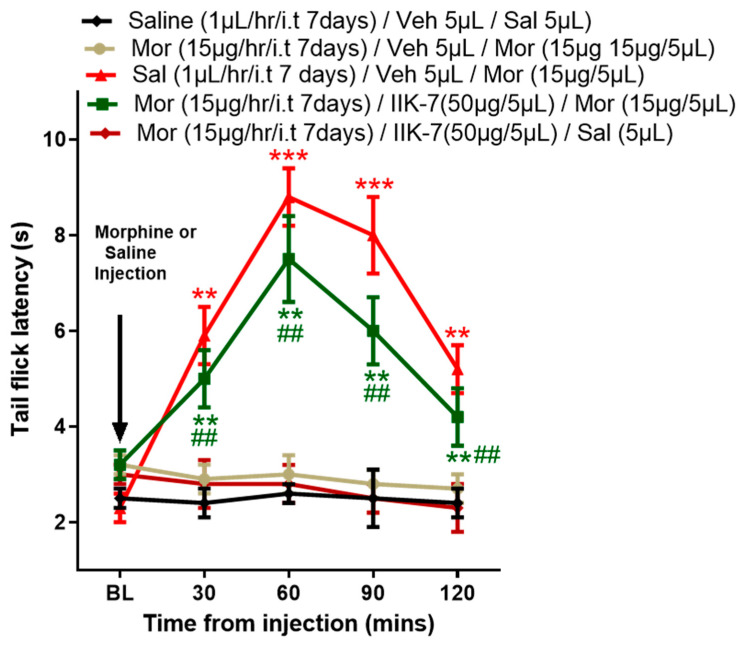
In rats tolerant to MOR, IIK7 restores the antinociceptive action of MOR. On Day 7, following the i.t. infusion of either MOR or saline, the antinociceptive effects were assessed after the disconnection of the i.t. pump. The rats were given i.t. injections of either 50 μg of IIK7 or vehicle 3 h after the infusion was stopped. Thirty minutes later, they were given a 15 μg MOR challenge, and for 120 min, tail-flick latency was measured every 30 min. For at least six rats, all data are displayed as means ± SDs. The asterisks indicate statistically significant differences among the groups Sal/Veh/Sal compared to MOR/Veh/MOR, MOR/Veh/MOR compared to Sal/Veh/MOR, and MOR/Veh/MOR compared to MOR, IIK7/Saline. # indicates statistically significant difference between MOR/Veh/MOR and MOR/IIK7/Mor: **/## *p* < 0.01; *** *p* < 0.001. Six animals were included in each group (*n* = 6).

To evaluate the efficacy of IIK7 in restoring MOR’s analgesic effects in MOR-tolerant animals and delineate the underlying mechanism, fifty micrograms of IIK7 or vehicle (10% ethanol) were injected following a 3 h period after the discontinuation of the osmotic pump on Day 7 of the infusion, as illustrated in Figure 6. MOR’s antinociceptive action was evaluated 30 min post IIK7 or saline injection by a tail-flick test. Figure 7 illustrates that a 15 μg i.t. injection of MOR produced considerable antinociception in saline-administered animals but not in the rats with MOR tolerance. In MOR-tolerant rats, however, pretreatment with IIK7 significantly reversed the MAT and restored MOR’s antinociceptive effect (Figure 7). However, the administration of IIK7 alone did not induce any tail-flick test response (acute pain/noxious stimulus); however, the anti-acute nociceptive effect was observed when co-administered with MOR.

### 2.6. IIK7 Restores MOR Analgesic Effects in Mechanical Allodynia and Hyperalgesia in MOR-Tolerant Rats

**Figure 8:**
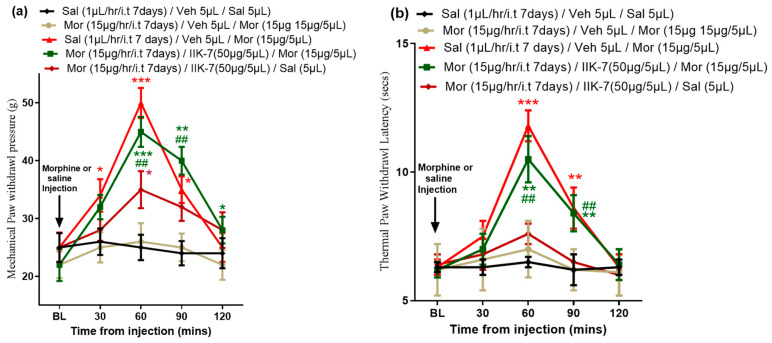
In PSNT rats tolerant to MOR, IIK7 effectively restores the antinociceptive action of MOR. On Day 7, following the conclusion of the i.t. infusion of either MOR or saline, the antinociceptive impact of the drug was assessed. The rats were given i.t. injections of either 50 μg of IIK7 or vehicle three hours after the infusion was stopped. Thirty minutes later, they were given a 15 μg MOR challenge, and for 120 min, (**a**) Mechanical paw withdrawal threshold and (**b**) Thermal paw withdrawal latency were measured every 30 min. For at least six rats, all data are displayed as means ± SDs. The asterisks indicate statistically significant differences among the groups Sal/Veh/Sal compared to MOR/Veh/MOR, MOR/Veh/MOR compared to Sal/Veh/MOR, and MOR/Veh/MOR compared to MOR, IIK7/Saline. # indicates a statistically significant difference between MOR/Veh/MOR and MOR/IIK7/MOR with the following *p*-values: * *p* < 0.05; **/## *p* < 0.01; *** *p* < 0.001. (*n* = 6 animals per group).

We followed the same procedure as the tail flick test to assess the efficacy of IIK7 in reversing MOR tolerance in the mechanical allodynia and thermal hyperalgesia tests. As shown in Figure 8a,b, saline-infused rats displayed a normal antinociceptive response 1 h post 15 μg of MOR i.t. injection in both mechanical allodynia (Figure 8a, 50 ± 5 g red curve) and thermal hyperalgesia (Figure 8b, 12 ± 0.9 s red curve), but not the MOR-tolerant rats (Figure 8a, 25 ± 5 g, yellow curve, and Figure 8b, 6 ± 0.5 s, yellow curve). The MAT was significantly reversed, and the analgesic response of MOR was successfully restored in MOR-tolerant rats when MOR was administered 30 min after pretreatment with IIK7 in both mechanical allodynia (Figure 8a, 45 ± 5 g green curve) and thermal hyperalgesia (Figure 8b, 10.8 ± 1 sec green curve). In addition, IIK7 single injection at a high dose can produce an antiallodynic and antihyperalgesic effect to partially recover the pain threshold similar to our previous report on PSNT rats. However, compared to PSNT rats, more PSNT rats have comparatively fewer antinociceptive effects.

### 2.7. IIK7 Suppresses Microglial Activation Induced by MOR in the Spinal Cord of PSNT Rats

**Figure 9:**
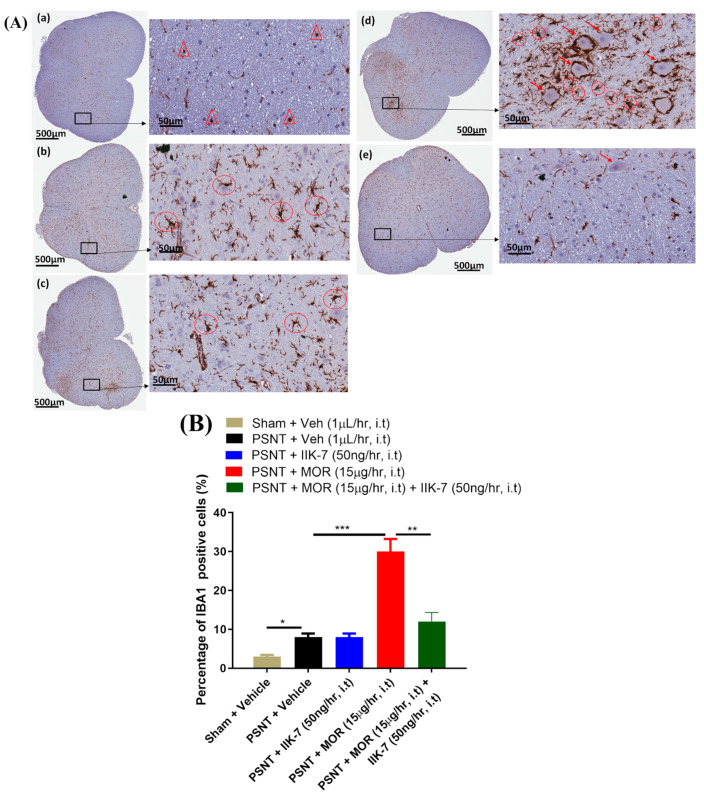
(**A**) Co-infusion of IIK-7 reduces microglial cell activation induced by morphine. Spinal cord samples were treated with IBA-1 and the nucleus was stained with DAPI (blue) seven days after the infusion of different combinations. Fluorescence microscopy was then used to acquire and blend the images. The shown sections are from rats that were infused with (**a**) sham/vehicle, (**b**) PSNT/vehicle, (**c**) PSNT/IIK-7, (**d**) PSNT/MOR, and (**e**) PSNT MOR + IIK-7 treatments. The dot-like red triangular structures represent resting microglia and chained morphology outlined in red circles indicates activated microglial cells. Red arrows indicate pericellular edema. (**B**) Impact of IIK-7 co-infusion on morphine-induced microglial cell activation. Active microglial cells were observed in the spinal cords of MOR-infused rats seven days post infusion compared with sham animals. IIK-7 markedly reduced microglial cell activation induced by morphine. Asterisks indicate statistically significant differences among the following comparisons: sham vs. PSNT + saline, PSNT + saline vs. PSNT+MOR, and PSNT MOR vs. PSNT + MOR + IIK-7. * *p* < 0.05; ** *p* < 0.01; *** *p* < 0.001 (*n* = 6).

We performed immunohistochemistry on spinal cord samples utilizing the microglial marker IBA-1 one week post drug infusion in PSNT rats and sham rats to examine the inhibitory impact of IIK7 on microglial cell activation triggered by PSNT and MOR continuous infusion. The shapes of IBA-1-positive cells in the sham group resembled little dots, indicating that the microglial cells were in an inactive state (Figure 9A(a)). Following the establishment of PSNT, IBA-1-positive cells exhibited marginally greater cell body hypertrophy in comparison to sham animals (Figure 9A(b)). The IIK7 ultra-low dosage infusion alone did not significantly affect microglial cell activity in PSNT animals compared to PSNT + Vehicle (Figure 9A(c)) animals. In comparison to the PSNT vehicle, the MOR infusion significantly exacerbated microglial activation, as evidenced by the ameboid morphology of the hyperactive cells (Figure 9A(d). IIK7, in combination with MOR, greatly reduced the activation of microglia triggered by MOR in comparison to MOR infusion alone (Figure 9A(e). In PSNT rat spinal cords, activated microglia were present, and MOR administration exacerbated these activations. Nonetheless, the co-infusion of IIK7 with MOR markedly diminished microglial cell activation by rectifying the pathogenic changes.

### 2.8. IIK7 Suppresses Spinal GFAP Activation Triggered by MOR in PSNT Rats

**Figure 10:**
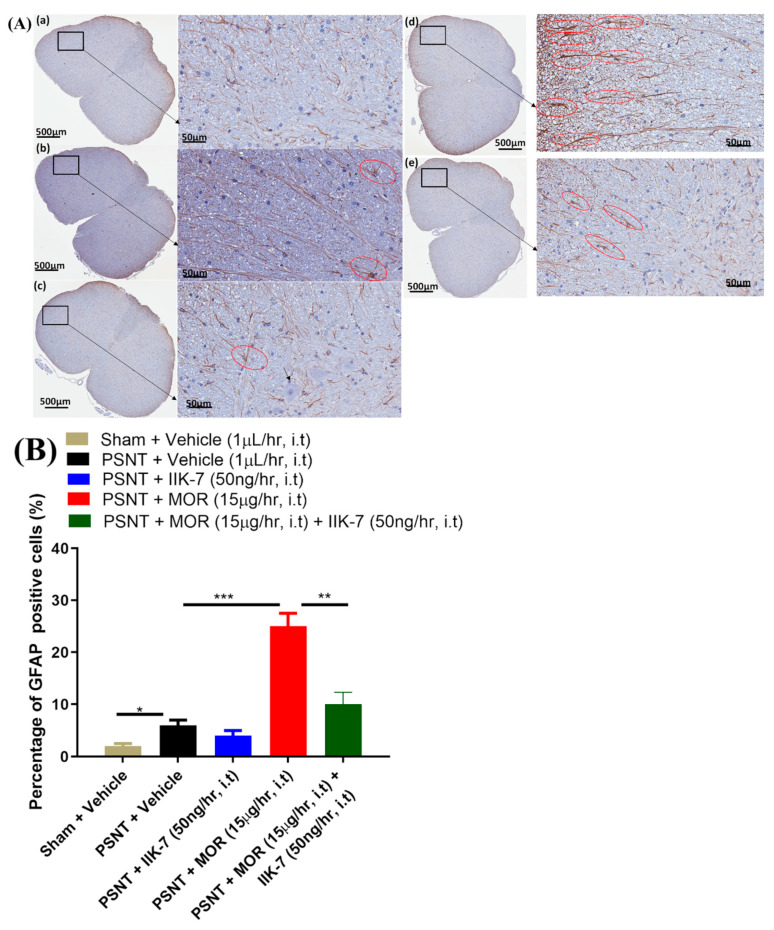
(**A**) In morphine-tolerant rat spinal cords, IIK-7 co-infusion decreases morphine-induced astroglial cell activation. Spinal cord slices were treated with GFAP and the nuclei were stained with DAPI stain (blue) seven days after the infusion of different combinations. Fluorescence microscopy was then used to acquire and blend the images. The shown sections are from rats that were infused with (**a**) sham/vehicle, (**b**) PSNT/vehicle, (**c**) PSNT/IIK-7, (**d**) PSNT/MOR, and (**e**) PSNT MOR + IIK-7. The stellate shapes of astrocytes with several intricate processes, outlined in red, were evident in GFAP-positive cells. The effects of 1× and 2× reduced astrogliosis. Arrows indicate pericellular edema. (**B**) The quantitative analysis of GFAP-positive cells. A marked positivity for IL-1β in the spinal cords of MOR-infused rats was observed 7 days post infusion compared with sham animals. The IIK-7 significantly attenuated morphine-induced astroglial cell activation. Asterisks indicate statistically significant differences among the following comparisons: sham vs. PSNT + Vehicle, PSNT + Vehicle vs. PSNT + MOR, and PSNT MOR vs. PSNT + MOR + IIK-7. * *p* < 0.05; ** *p* < 0.01; *** *p* < 0.001 (*n* = 6).

In NP, the astrocyte activation can be regarded as an important sign of oxidative stress. The PSNT group had slightly more positive GFAP cells than the animals in the sham group (Figure 10A(b)) according to the immunofluorescence results. No substantial morphological changes were evident in IIK7-infused rats in comparison to vehicle-infused rats (Figure 10A(c)). In the MOR-infused spinal cord slices, GFAP-positive cells, which generally resembled astrocytes with several long processes, were clearly evident (Figure 10A(d)). However, IIK7 co-infusion markedly reduced GFAP activation (Figure 9A(e)).

## 3. Discussion

Our results show that IIK7, which is a melatonin type 2 receptor agonist, greatly lowers the activation of microglia and astrocyte cells caused by MOR infusion. The co-infusion of IIK7 reduced the increase in PIC levels induced by MOR and normalized Nrf2/HO-1 levels in rats with MAT. Consequently, IIK7 effectively reduced the progression of MAT. This research may offer a novel approach by suppressing microglial and astrocyte activation, thereby enhancing the clinical analgesic effectiveness of MOR.

Chronic MOR infusion resulted in antinociceptive tolerance, consistent with our prior research, and a MOR challenge significantly diminished the antinociceptive effect in MOR-tolerant rats [35,36,37]. In this study, the infusion of the melatonin receptor agonist IIK7 greatly attenuated MAT. Additionally, in the spinal cord, chronic MOR-infused animals showed increased microglial activity and astrocyte expression; both effects were countered by IIK7 co-infusion. In the current investigation, we discovered that IIK7 co-infusion not only attenuated MAT but also restored the antinociceptive effect of MOR in MOR-tolerant rats.

Neuroinflammation is a significant factor in the development of MAT. It is linked to glial-cell and astrocyte activation and elevation of reactive oxygen species (ROS) or PICs [38]. The involvement of microglia in neuroinflammation related to the development of MOR tolerance was investigated by immunocytochemistry. It has been demonstrated that microglial and astrocyte activation is crucial for the release of cytokines [39]. Activated microglia show the elevated expression of the PICs TNF-α, IL-1β, and IL-6 following chronic exposure to MOR [40,41]. These lead to the release of neurotoxic chemicals, including neurotrophic factors, nitric oxide, chemokines, PICs, and ROS [42]. Signaling molecules releasing from injured neurons, including cytokines and chemokines, may initiate the activation of glial cells [43].

The chronic administration of MOR in rats activates neuronal protein kinase Cγ, resulting in the phosphorylation of opioid receptors and increased communication between neurons and glia; these interactions contribute to MAT [44]. It is well known that activated glia can lead to the development of MAT [19]. MOR treatment for prolonged durations results in (a) glial-cell and astrocyte activation [45], (b) the upregulation of PIC expression [46], and (c) neuroinflammation [35]. The inhibition of glial cell activation is known to restore the analgesic effect of MOR in MOR-tolerant rats [17]. Activated microglia are subsequently activated by astrocytes, thus developing inflammatory pain [47].

Microglia comprise 5–10 percent of all glial cells in the CNS, serving roles in immune surveillance and the detection of harmful stimuli. Previous findings indicate that MAT elevates the expression of PICs in microglia [48]. The administration of microglial cell inhibitors reversed morphine tolerance by inhibiting p38 MAPK phosphorylation in microglia [49,50]. Microglial cell activation can trigger the release of brain-derived neurotrophic factor, subsequently enhancing the expression of NMDA receptors in neurons. Recent research indicates that astroglia are significant contributors to the pathogenesis of neuropathic pain and inflammation. Garrison et al. had demonstrated a connection between astrocyte activation and heightened pain hypersensitivity [51]. Moreover, several chronic pain conditions, including spinal cord injury, nerve trauma, and prolonged opioid exposure, consistently show astrogliosis, indicated by increased GFAP expression [17,52,53]. No study has, to our knowledge, investigated IIK7′s efficacy in minimizing the activation of microglia and astrocytes and its impact on PIC expression in the NP model with MOR tolerance.

Our study aimed to investigate the impact of ultra-low-dose co-infusion of IIK7 on the attenuation of MOR tolerance in PSNT rats, utilizing measurements of PWT in response to mechanical stimuli, PWL in response to thermal stimuli, and tail-flick tests. Additionally, we assessed the effectiveness of IIK7 in reducing PIC levels, as well as the expression of the antioxidant genes NRF2/HO-1 and the primary mediators of MAT microglia and astrocytes. Moreover, we assessed the effectiveness of a high dose of IIK7 in reversing pre-existing MOR tolerance. The co-infusion of IIK7 at a dosage of 50 ng/h along with MOR notably reduced MOR’s analgesic tolerance in PSNT rats; the infusion of IIK7 at a dose of 50 ng/h alone demonstrated no significant analgesic effect when compared to vehicle-infused PSNT rats. Furthermore, we found that the i.t. administration of IIK7 at a dosage of 50 μg can reverse pre-established MAT. We found that MOR tolerance upregulated TNF-α, IL-1β, and IL-6 expression similar to the existing literature (Figure 5a–c) [54]. It is interesting to note that IIK7 was shown to prevent the generation of cytokine expression activated by chronic MOR i.t. infusion (Figure 5).

Our previous research evidence suggests that a low dose of melatonin (3 μg/h, i.t.) along with MOR lowers their tolerance and partially restores their sensitivity to MOR in both naïve and neuropathic rats [55]. However, a higher dose (6 μg/h, i.t.) does not influence tolerance [55]. This varying effect might relate to melatonin’s physiological levels. As Irina et al. found, a lower dose of melatonin (0.3 mg orally) effectively restored nocturnal physiological levels in insomnia patients, resulting in better sleep than a higher dose (3 mg orally) [56]. Similarly, Lewy et al. reported that melatonin doses between 20 and 300 μg successfully normalized physiological levels and aligned the free-running circadian rhythm in blind individuals [57]. Our i.t. infusion model skipped the liver’s metabolism, mimicking the levels of melatonin in the cerebrospinal fluid. These findings imply that combining low-dose melatonin or its agonists with MOR in clinical patient-controlled analgesia via the spinal route could effectively manage pain. Further research is needed to explore the concentration achieved through continuous i.t. infusion and the physiological and therapeutic melatonin levels in the CSF.

Researchers typically assessed higher melatonin dosages (up to 300 mg, orally) for their antinociceptive effects, tying their actions to either MT2 or opioid receptors [30,58,59]. Tututi et al. found that the intravenous administration of melatonin (up to 100 μg) and orally (37.5–300 mg/kg) to rats with spinal nerve ligation improved allodynia through the involvement of opioid and MT2 receptors [60]. This was because melatonin affected both MT2 and opioid receptors. Lin et al. discovered that giving 100 mg/kg of melatonin intraperitoneally reduced both thermal and mechanical allodynia [61]. This suggests that both MT2-dependent and independent pathways are involved.

This study report demonstrates that an ultra-low dose of IIK7 (50 ng) effectively delays MOR tolerance development, though the rats did not show any efficacy in minimizing neuropathic pain. However, a high dose of IIK7 (50 μg) is highly effective in the restoration of MOR’s antinociceptive effect in MOR-tolerant rats. Compared to melatonin (50 μg i.t.) in our previous report, IIK7 (50 μg i.t.) displayed a more pronounced restoration of MOR’s antinociceptive effects [62]. This may be ascribed to the subsequent factors: research indicates that MOR administration decreases circulating melatonin levels and downregulates the melatonin receptor MT1 while MT2 remains unaffected [63,64]. Moreover, MT1 is crucial in the formation of MOR tolerance. It accomplishes this by inhibiting the cAMP signal transduction cascade, hence reducing PKA activity and CREB phosphorylation [65]. Therefore, MT-2 receptor agonists may represent a more suitable option for reinstating MOR tolerance compared to MT-1 receptor agonists. Melatonin can reduce MOR tolerance via many routes. Melatonin can reduce NLRP3 inflammasome activity, which initiates inflammatory responses and oxidative stress associated with MOR tolerance [64]. Melatonin is recognized for its ability to suppress the functions of PKC and NMDA receptors in the spinal cord; these play a role in the onset of MOR-induced hyperalgesia and tolerance [66], and melatonin aids in reversing mitochondrial malfunction and excessive oxidative stress, which are critical contributors in the development of MOR tolerance. By targeting these pathways, a non-nociceptive dose of melatonin can significantly diminish MOR tolerance and enhance its analgesic efficacy. The MT2 receptor is crucial in regulating oxidative stress [67]. Melatonin mitigates oxidative stress by interacting with MT2 receptors and altering numerous cellular pathways [30]. This involves reducing levels of protein carbonyl (PCO) and malondialdehyde (MDA), indicators of oxidative damage, while increasing the total antioxidant capacity (TAC) [30]. Therefore, we hypothesize that the co-infusion of a non-nociceptive dose of 50 ng of IIK7 with MOR may mitigate MOR tolerance by stimulating antioxidant pathways essential for regulating oxidative stress and inflammation. This entails diminishing oxidative stress and regulating inflammatory responses, which are critical elements in the progression of MOR tolerance. On the other hand, a high dose of 50 µg IIK7 can result in a rapid activation of the MT-2 receptor, which can activate the antioxidative enzymes CAT, SOD, and GPX, hence mitigating oxidative stress and inflammation linked to MOR tolerance [68].

The mRNA levels of Nrf-2 and HO-1 were assessed in the spinal cord to elucidate the mechanisms underlying MAT attenuation. MLT and its agonists are recognized for their ability to stimulate the expression of neuroprotective genes associated with anti-inflammatory functions, specifically Nrf-2 and HO-1 [69,70]. The findings indicate that IIK7 markedly enhances the mRNA levels of Nrf2 and HO-1 in PSNT rats when used in combination with opioid MOR. The current literature suggests that the activation of Nrf2 and HO-1 may enhance the analgesic efficacy of morphine through various mechanisms. Sulforaphane activates Nrf2, enhancing the antiallodynic and antihyperalgesic effects of MOR. The increased expression of mu-opioid receptors and HO-1 is known to improve the antinociceptive effects of MOR [71]. Melatonin and its agonists demonstrate multiple pleiotropic effects, including anti-apoptotic signaling [72], neuroprotection, and the enhancement of synaptic plasticity, all of which contribute to reductions in the MAT [73,74].

Following nerve injury, microglial cell activation and the subsequent release of PICs are known to activate immune cells such as macrophages and lymphocytes, serving as a neuroprotective mechanism [75]. The immune cells’ migration to the injury site is a key inflammatory feature in the innate immune response [75]. NP and MAT are acknowledged for their contributions to neuroinflammation and immune cell activation in the spinal cord [76,77,78,79]. The results indicate that IIK7 co-infusion markedly decreases MOR-induced glial cell activation and immune cell infiltration in the spinal cord. This study demonstrated notable findings concerning the influence of IIK7 on the antinociceptive effects of MOR and MAT in PSNT rats. Additional research is required to thoroughly clarify the mechanisms underlying MAT attenuation and reversal.

## 4. Materials and Methods

### 4.1. Regents

Morphine and dimethyl sulfoxide (DMSO) were purchased from Sigma-Aldrich (St. Louis, MO, USA). IIK7 was acquired from Santa Cruz (Dallas, TX, USA). IIK7 and MOR were formulated in a 4% DMSO solution. DMSO exhibited no significant antinociceptive or side effects on rats at the dosages utilized in our study [80,81]. The Iba1 antibody was procured from Santa Cruz Biotechnology (1022-5) Sc-32725. The GFAP antibody was procured from Atlas Antibodies, HPA056030 (Stockholm, Sweden).

### 4.2. Animals

The Animal Care and Use Committee of the National Defense Medical Center has assessed and approved our study methods, adhering to the standards of the National Institutes of Health Guide for the Care and Use of Laboratory Animals (IACUC107-019). We accommodated male Wistar rats, aged 7 weeks (BioLASCO Taiwan Co., Ltd., Taiwan), two animals per cage with soft bedding, adhering to a 12 h light/dark cycle and providing them with free access to food and water. We enacted all means to diminish the population of animals and mitigate their suffering.

### 4.3. Developing an Animal Model for Neuropathic Pain and i.t. Catheterization

The left sciatic nerve in the PSNT rats was meticulously exposed to the mid-thigh region. A prolene 7-0 ligature was then inserted at the midline of the nerve, situated just cranially to the branch that innervates the biceps femoris muscle. This resulted in a ventrocranial transection of half the nerve, reaching the ligature. The left sciatic nerve was gently pulled in the sham group, after which the wound was closed with sutures. We evaluated the PWT for mechanical stimuli in the sham group and the PSNT group prior to PSNT surgery and 7 days post surgery. The study excluded rats exhibiting signs of motor impairment. On Day 7 following PSNT, an i.t. catheter/osmotic pump was implanted under 2% to 2.5% isoflurane anesthesia [81,82]. We fabricated the i.t. catheter utilizing the method previously described [48]. Each rat’s cisternal membrane was meticulously perforated to implant a polyethylene catheter (PE 10 tubing, 8.0 cm), which was then threaded caudally to access the lumbar expansion of the spinal cord. This catheter was connected to the osmotic pump (flow rate 1 μL/h; ALZET, Cupertino, California) and randomly divided into the following five groups with six animals per group: sham-operated vehicle, PSNT/vehicle, PSNT/IIK7 50 ng/h, PSNT/MOR 15 μg/h, and PSNT/MOR 15 μg + IIK7 50 ng/h. The randomization sequence was built using a computer-made random number table. To evaluate the effectiveness of IIK7 in reversing established MAT, two i.t. catheters were implanted for drug administration. One was connected to an osmotic pump for MOR or saline infusion. On the seventh day, the osmotic pump was disconnected and injected with 10 μL saline, followed by the injection of 50 μg of IIK7 or vehicle administration via the second catheter. Subsequent to drug administration, the catheter was irrigated with 15 μL of saline. After three hours, 15 μg of MOR or saline was administered, and the antinociceptive effect was evaluated using the tail-flick test, mechanical PWT, and thermal PWL. The animals were later divided in to groups in the sequence listed above by an unbiased researcher not involved in the study. The assignment of saline or III7 or MOR or MOR + IIK7 treatment was randomized to eliminate any systematic bias. During the experiment, the researchers were blinded to the group allocations to reduce bias. The outcome evaluation was conducted by blinded researchers to ensure unbiased results. The data analysis was performed by a statistician unaware of the group assignments to maintain impartiality.

### 4.4. Antinociception Test

A hot water immersion test at 52 ± 0.5 °C was utilized to assess tail-flick delay. The baseline delay was around 2 ± 0.38 s, with a cutoff duration set at 10 s. We secured the rats in plastic restrainers for the tail-flick assay and to assess medication effects.

### 4.5. Evaluation of Thermal Hyperalgesia and Mechanical Allodynia

We evaluated the sensitivity of the rat paw plantar region with the Dynamic Plantar Anesthesiometer (Ugo Basile, Comerio, Italy). Rats were separated in a container equipped with a wire-webbed bottom, and rats were allotted a 15 min adaptation period prior to each test. A paw withdrawal reaction was elicited by exerting an escalating tension with a metal filament (0.5 mm in diameter with a blunt end) at the mid plantar surface. The force was incrementally increased from 1 g to 50 g in 1 g increments over a duration of 20 s, then sustained at 50 g for an extra 10 s. We defined the tactile threshold as the force required to elicit reflexive withdrawal of the hindpaw, recording it as the average of three measurements taken at one-minute intervals.

Thermal hyperalgesia was evaluated with a Hargreaves radiant heat apparatus (7371; Ugo Basile, Comerio, Italy; infrared setting 80). Rats were housed in an enclosure, allowing a 15 min acclimatization interval between the tests. A plastic cage was placed above the testing apparatus, positioning a moveable infrared heat source just under the plantar surface of the hindpaw. Upon activation, the gadget generated a continuous emission of heat to the plantar region. This heat-elicited paw withdrawal reaction was automatically recorded by a timer. A 5 min interval was given between each test, and the mean latency was measured for each paw with three measurements. The reference threshold ranged from 8 to 10 s in normal rats, with a limit of 22 s implemented to avert tissue damage.

### 4.6. Real-Time Quantitative Polymerase Chain Reaction (RT-qPCR)

Seven days post pump installation, we sacrificed the rats, and spinal cords were collected. qPCR was performed by following the previously reported protocols [83]. Total RNA was isolated using TRIzol reagent (Takara, Japan) and subsequently reverse-transcribed into cDNA following the outlined procedures: 25 °C for 5 min, 42 °C for 30 min, and 85 °C for 5 min. The reverse transcription reaction system comprised 10 μL of RNase-free ddH_2_O, 2 μL of 5× gDNA digester buffer, 1 μL of gDNA digester, 1 μg of total RNA, and 10 μL of 2× Hifair^®^ II SuperMix plus. The reaction system comprised 1 μL forward primer, 1 μL reverse primer, 10 μL 2× mix, 7 μL H_2_O, and 1 μL cDNA, utilized in RT-qPCR to assess the mRNA expression levels of target genes on the 12K fluorescence RT-qPCR apparatus (ABI, LA, USA). The primer sequences are enumerated in Table 1. GAPDH functioned as a reference control. The data were computed using the 2–DDCt technique. The data have been presented as means and standard deviations.

### 4.7. Evaluation of Pro-Inflammatory Cytokines

We isolated the lower lumbar expansion (L4-6) of the spinal cord after behavior experiments and then extracted the homogenates using RIPA lysis buffer (150 mmol/L NaCl, 0.5% sodium deoxycholate, 5 mmol/L EDTA, 0.5% NP-40, 50 mmol/L Tris-HCl, pH 6.8), supplemented with a commercial protease and phosphatase cocktail (Applygen, Beijing, China). The tissue homogenates were centrifuged at maximum speed for 15 min at 4 °C. A BCA protein assay (Thermo Pierce, Rockford, IL, USA) was performed to ascertain the protein concentrations. The concentrations of TNF-α, IL-1β, and IL-6 in tissue lysates were assessed using commercial ELISA kits (R&D Systems, Minneapolis, MN, USA) in accordance with the manufacturer’s guidelines. We have represented the data as picograms per milligram of protein.

### 4.8. Immunofluorescence Studies

Subsequent to the behavior tests, the rats were humanely euthanized utilizing isoflurane anesthesia (Abbott Laboratories Ltd., Queenborough, Kent, UK). The lumbar region of the spinal cord was meticulously excised and subsequently fixed in a 4% formaldehyde solution for 8 h, followed by fixation in 30% sucrose for an additional 8 h. The samples were subsequently paraffin-embedded and sectioned to a thickness of 10 µm. The slides were incubated for 12 h at 4 °C with the corresponding antibody. The slides were then stained with DAPI (Sigma-Aldrich, St. Louis, MO, USA) for one hour and scanned with a Pannoramic 205 FLASH II slide scanner. The scanned photos were processed using the case viewer software. Quantitative analyses were performed using the ImageJ program (https://imagej.net/, accessed on 22 November 2024).

### 4.9. Statistical Analysis

The data have been expressed as means ± SDs. All graphical representations and statistical calculations were aided by GraphPad Prism version 6.01. Two-way ANOVA, Tukey’s multiple comparisons test, and Student’s *t*-test were used to analyze statistical significance. The sample size of six animals per group aligned with comparable studies in the literature that had identified significant effects in MOR tolerance animal models [84]. Prior research indicated that this sample size was enough for identifying alterations in gene expression and behavioral results. For example, Yadlapalli et al. [85] have demonstrated that a group size of six is sufficient for detecting analgesia, tolerance, and the mechanism of action of MOR across multiple pain modalities in rats.

## 5. Conclusions

Our study results indicate that the co-infusion of ultra-low-dose 50 ng IIK7 can attenuate MAT development. Furthermore, IIK7 effectively reverses pre-existing MAT with a single injection of 50 μg. It was found that IIK7 co-infusion decreased the levels of PICs and increased the levels of Nrf-2 and HO-1 mRNA expression in the spinal cord. More importantly, the co-infusion of IIK7 greatly reduced the activation of MOR-induced glial cells and astrocytes in the spinal cords of PSNT rats. This study established a foundation for the potential clinical application of melatonin or IIK7 in the management of MAT in patients experiencing chronic pain. We need to conduct more research to confirm the potential role of the MT-2 receptor and its impact on MAT and oxidative stress in PSNT rats for their potential role in clinical neuropathic pain management.

## Figures and Tables

**Table 1:** Primer sequences for RT-qPCR.

Gene Name	Sequence of Primers
Nrf-2	Forward: CCCATTGAGGGCTGTGAT Reverse: TTGGCTGTGCTTTAGGTC
HO-1	Forward: GCATGTCCCAGGATTTGTCC Reverse: GGTTCTGCTTGTTTCGCTCT
GAPDH	Forward: ACTCCCATTCTTCCACCTTTG Reverse: CCCTGTTGCTGTAGCCATATT

## Data Availability

The data presented in this study are available within the article.
